# Chicago Veterinary Society

**Published:** 1897-01

**Authors:** Lawrence Campbell

**Affiliations:** Secretary


Secretary.

### CHICAGO VETERINARY SOCIETY.

A meeting was called to order on the evening of December 10, 1896, by the President, Dr. R. G. Walker. Roll-call showed twenty-seven members present. The minutes of the previous meeting were read and approved.

The President made a short address, in which he asked all members to be present hereafter promptly at 8 P.M., so that the meeting may come to order and the Secretary finish the roll-call by a quarter past. He also made a special request that all the members use their efforts with their representatives in support of the "Army Veterinary Bill" for the regulation of the positions and pay of veterinary surgeons in the United States Army.

The committee appointed to draft the by-laws and constitution of this

ARMY MEDICAL LIBRARY WASHINGTON, D. C. U. S.

Society were, on motion, discharged. Moved and seconded that the Secretary be appointed to provide copies of the by-laws and constitution, and he shall also be authorized to secure any other needed stationery or printed matter. One of the members suggested that very likely some one of the instrument-houses would gladly print the by-laws for the Society if the house would be permitted to place their advertisement on the cover. Motion by Dr. Gysel, seconded by Dr. Johnson, that the Society pay for its own printing, and that no advertisements be permitted. The President, Dr. Walker, being suddenly called away, the chair was taken by Dr. Dyson, First Vice-President.

The next order of business was the reading of a paper on "Dentistry," by Dr. L. A. Merillat. The paper was a general one, dealing with no particular disease. The author censured veterinary surgeons in general as veterinary dentists, which condemnation grows out of the fact that too little care and study is applied to this subject, and how much better operators many unqualified men are than college graduates, who should be the leaders in all branches of the profession. The paper gradually led up to the special diseases of the teeth, with which special diseases Dr. Merillat very kindly offered to deal at the next regular meeting. At the close of the paper he stated that in his opinion there was hardly a horse that had not *some* irregularity of the teeth which a proper operation could improve, and that every veterinary surgeon should be thoroughly competent to do such operation when called upon. The discussion following this paper was very interesting. The question of price for an ordinary operation of floating was brought up, the general sentiment being that the fee should be $3.00. Dr. Merillat thought that when a horse was brought to his hospital $2.00 was a sufficient charge for fifteen or twenty minutes' work on a horse's mouth. Drs. Sayre and E. L. Quitman said they charged $3.00, and tried always to maintain that price even at the hospital. This discussion was conducted by Drs. Merillat, E. L. Quitman, Sayre, P. Quitman, Robertson, and Campbell. The subject of "Bishoping" was brought up by Dr. Hawley, who wanted to know if veterinary surgeons should refuse to bishop horses' teeth when requested to do so by horse dealers. This called forth quite a little discussion. A few of the members present said they had formerly made a practice of bishoping; still, it was years ago, and before they knew better; but that now they condemned the practice. Dr. Hawley stated that many horse buyers for some unknown reason would not purchase horses over six years old, that they seemed to think that as soon as a horse was seven years of age he was on the down-grade of life. Consequently there was no market for an eight- or nine-year-old horse, and many large sums of money were lost to the farmer and dealer through the buyer's opinion, while we knew that a horse was in his prime at from eight to twelve or even fourteen years, providing his care had been good. For this reason he thought that to bishop an eight-year-old horse to a six was not a swindle and not an imposition upon the buyer; that the horse is just as serviceable to the last owner as a six-year-old, and more money could be gotten for the animal, which would be a greater benefit to the stock raiser as well as to the dealer. He would not bishop an old animal, as he would not consider this honest. This subject was discussed by Drs. Hawley, Sayre, Robertson, E. L. Quitman, and Campbell. Dr. Merillat asked if anyone had ever filled a dog's teeth. Drs. Sayre and E. L. Quitman replied they had had some experience in this line. Dr. Merillat said he had been called upon to sharpen up the teeth of an old "bull dog," to aid in his fighting qualities. On motion and vote, the discussion was closed.

A proposal and motion were made by Dr. A. H. Baker, seconded by Dr. E. L. Quitman (voted and carried unanimously by the Society), "that a notice be sent to the City Press Association exonerating one of the Society's members for the shooting and killing of one Joseph Kramer, as follows:

WHEREAS, One of our members, Dr. A. C. Worms, has had the misfortune to accidentally shoot and kill a thief, Joseph Kramer, caught in the act of stealing the blanket off his horse, while attending a case in the practice of his profession, and has been held to the grand jury by the coroner's jury for murder; be it

*Resolved*, That this Society from the evidence at hand exonerates Dr. Worms from intentional criminality in this unfortunate accident.

Dr. Worms stated to the Society that he was running after the thief with his pistol in his hand, when it was accidentally discharged. That he had twice called to the thief to halt before the discharge, although at the time of the discharge the thief did not have possession of the blanket, which had been dropped. Dr. Worms was 125 feet behind Kramer in a rough alley, and as it was 10.30 at night and very dark, he was not aware that the bullet had hit Kramer, who continued to run for one and a half blocks when he fell backward, dead. No more shots were fired, this being good evidence that Dr. Worms did not intend to shoot Kramer. There were no witnesses to the shooting. Dr. Worms remained by the corpse until the police came. The coroner's jury held him on his own evidence, this being that the shot was not fired until after the blanket had been dropped by Kramer. Dr. Worms was allowed to sleep at home under the care of an officer until the following day, when he was released on $5000 bail. Many strangers have called on Dr. Worms since the shooting and are willing to testify at the trial that they were well acquainted with Joseph Kramer, and, although only twenty years of age, that he had been a sneak-thief for ten years.

Motion by Dr. Hughes, seconded by Dr. E. L. Quitman, that at our next regular meeting, January 14, 1897, the members of this Society hold a banquet at the Sherman House, and that the regular programme be carried out at the banquet table. Voted and carried.

Motion by Dr. Hughes, seconded by Dr. E. L. Quitman, that the Secretary be instructed to send invitations to all the members of the Society to attend this banquet.

No new members presenting themselves, adjournment was in order.

LAWRENCE CAMPBELL,
Secretary.

A meeting of the Iowa State Veterinary Medical Association will be held on January 13 and 14, 1897. Papers on the following subjects will appear on the programme: "The Relation of the Veterinary Profession to the Public Health," "Parasitic Diseases in Sheep," "Duties of the State Veterinarian in Iowa," "Azoturia," "Fungoid Growths," "On the Contagium of Tuberculosis," "The Thermo-cautery in Veterinary Practice," "Serum-therapy in Hog-cholera" by Dr. Peters, and "Experiments with Barium Chloride in the Treatment of Colic."

